# Genetic polymorphisms of 44 Y chromosomal genetic markers in the Inner Mongolia Han population and its genetic relationship analysis with other reference populations

**DOI:** 10.1080/20961790.2020.1857509

**Published:** 2021-02-11

**Authors:** Xiaoye Jin, Guohui Xing, Chunhua Yang, Xingru Zhang, Wei Cui, Chong Chen, Bofeng Zhu

**Affiliations:** a Key Laboratory of Shaanxi Province for Craniofacial Precision Medicine Research, College of Stomatology, Xi’an Jiaotong University, Xi’an, China; b College of Forensic Science, Xi’an Jiaotong University Health Science Center, Xi’an, China; c Clinical Research Center of Shaanxi Province for Dental and Maxillofacial Diseases, College of Stomatology, Xi’an Jiaotong University, Xi’an, China; d People’s Hospital of Arong Banner, Hulun Buir City, China; e Multi-Omics Innovative Research Center of Forensic Identification; Department of Forensic Genetics, School of Forensic Medicine, Southern Medical University, Guangzhou, China

**Keywords:** Forensic sciences, forensic genetics, Y chromosomal, STR, InDel, Inner Mongolia Han, male differentiation

## Abstract

Y chromosomal genetic markers in the non-recombining region are commonly used for human evolution research, familial searching, and forensic male differentiation since they strictly follow paternal inheritance. Y chromosomal short tandem repeats (Y-STRs) possess extraordinarily advantages in forensic applications because of their high polymorphisms and special genetic pattern. Here, we assessed the genetic diversities of 41 Y-STRs and three Y chromosomal insertion/deletion (Y-InDels) loci in the Chinese Inner Mongolia Han population; besides, genetic differentiation analyses among the studied Han population and other previously reported populations were conducted based on 27 same Y-STRs. Totally, 425 alleles were observed in 324 Inner Mongolia Han individuals for these Y-markers. Gene diversities of these Y-markers distributed from 0.0306 to 0.9634. The haplotype diversity and discriminatory capacity of these Y-markers in the Inner Mongolia Han population were 0.9999 and 0.98457, respectively. Haplotype resolution comparisons of different Y-marker groups in the studied Han population revealed that higher haplotype resolution could be achieved for these 44 Y-markers. Population genetic analyses of the Inner Mongolia Han population and other reference populations demonstrated that the studied Han population had relatively closer genetic affinities with Northern Han Chinese populations than Southern Han and other minority groups. To sum up, these 44 Y-markers can be utilized as a valuable tool for male differentiation in the Inner Mongolia Han population.

## Introduction

Y chromosomal molecular genetic markers in the non-recombining region are paternally inherited, and therefore are widely employed for exploring human paternal evolution history [1,2], investigating genealogical relationship [3,4] and forensic male identification [5–7]. Among various genetic markers, Y chromosomal STRs show extraordinary value in forensic application due to their high polymorphisms and paternal inheritance patterns. Till now, an army of Y-STR kits have been developed for forensic practices [8–12]. However, previously used Y-STR sets have difficulty in differentiating closely and distantly related male relatives [5]. In a previous study, Ballantyne et al. [13] assessed mutation rates of 186 Y-STRs in a number of father-son pairs and found 13 rapidly mutating Y-STRs which could be used for distinguishing close and distantly related males better. Some of these rapid mutating Y-STR loci have begun to be added into existing Y-STR panels. For example, the PowerPlex^TM^ Y23 System (Promega Corporation, Madison, WI, USA) incorporated six novel Y-STRs into an extant multiplex system of 17 Y-STRs of the Applied Biosystems AmpFℓSTR^TM^ Yfiler^TM^ kit (Applied Biosystems, Foster City, CA, USA), and the novel panel demonstrated higher haplotype diversity and discriminatory capacity than other panels (minimal haplotype, Yfiler and PowerPlex Y12) [8]. However, more highly polymorphic Y-STRs are indispensable for the transition from male lineage differentiation to male individual identification.

To further improve haplotype discriminatory capacity among the male lineage, nine rapidly mutating Y-STRs (DYF387S1, DYF404S1, DYS449, DYS518, DYS570, DYS576 and DYS627), 13 new Y-STRs/InDels (rs199815934, DYS388, DYS447, DYS444, DYS645, rs771783753, rs759551978, DYS643, DYS557, DYS522, DYS596, DYS593 and DYS549) and a multi-copy locus DYS527 were used to construct a novel multiplex system on the basis of the loci in the Applied Biosystems AmpFℓSTR^TM^ Yfiler^TM^ Plus kit (Applied Biosystems). The novel kit, SureID^®^ PathFinder Plus (Health Gene Technologies, Ningbo, China), can simultaneously amplify 41 Y-STRs and three Y-InDels in a single well *via* the six fluorescent dye-labeled technology, which might possess better forensic potential for male individual identification. Three Y-InDels in this kit could be used as slowly mutating loci for familial searching. Besides, they also could enhance discriminatory capacity of the kit to some degree. In the previous research, Fan et al. [14] performed the developmental validation of the kit and found that it was good for forensic DNA database construction, male differentiation and familial searching. Besides, they also investigated genetic distributions of Y-STRs in the kit in Zhejiang Han population and found that the kit provided higher forensic application values than other kits. Some studies pointed out that Han populations in different regions showed different genetic structure, especially for South and North Han populations [15,16]. Therefore, the forensic efficiency of the novel kit in other Han populations should be evaluated.

Inner Mongolia is located in North China region. Many ethnic groups live in the Inner Mongolia region. Among these populations, Han population is the largest population in the Inner Mongolia region. To further evaluate forensic values of the SureID PathFinder Plus system in Chinese populations, we investigated the genetic distributions of 41 Y-STRs and three Y-InDels in the Inner Mongolia Han population. Furthermore, phylogenetic relationship analyses between the studied Han population and other published populations [9,17–49] were further conducted based on 27 Y-STRs of Yfiler Plus set.

## Methods

### Sample information

Bloodstain samples from 324 unrelated healthy Han male individuals residing in the Inner Mongolia Autonomous Region, China were collected. The studied individuals must live in the Inner Mongolia Autonomous Region, China, for more than three generations. All participants provided their written informed consent. The research protocol was approved by the Ethics Committee of Xi’an Jiaotong University Health Science Center (No. 2019-1039).

### Y-STR typing

Multiplex PCR of 41 Y-STRs and three Y-InDels was conducted according to the description of the SureID PathFinder Plus kit (Health Gene Technologies). In short, a 1 mm sample disc was added to the PCR cocktail consisting of 12.5 µL PathFinder Plus Master Mix, 6.25 µL PathFinder Plus Primer Mix, and 6.25 µL DNase/RNase-Free H_2_O. Amplification reaction of each sample was performed on the GeneAmp PCR System 9700 instrument (Applied Biosystems) according to recommended parameters by the company. Next, 1 µL PCR product/PathFinder Plus Allelic Ladder Mix was added to 8.5 µL HiDi formamide and 0.5 µL SIZE-580. The mixture was detected by the ABI3500xL Genetic Analyzer (Applied Biosystems). Genetic typing of 44 Y-markers was determined by GeneMapper ID-X Manager software (Applied Biosystems). Control DNA 9948 and DNase/RNase-Free H_2_O were used as positive and negative controls, respectively.

Furthermore, 10 null alleles at three single-copy Y-STRs (DYS389II, DYS447, and DYS448) and three multi-copy Y-STRs (DYF387S1, DYS527, DYF404S1) were observed in some individuals. These individuals were again detected by the Yfiler™ Platinum PCR amplification kit (Applied Biosystems). The experimental process was conducted according to the kit’s instruction.

### Statistical analysis

Allele frequencies and gene diversities (GDs) of 41 Y-STRs and three Y-InDels in the Inner Mongolia Han population were calculated by the STRAF online programme [50]. Haplotype frequencies of these loci were estimated by Arlequin software version 3.5 [51]. Haplotype match probability (HMP), discriminatory capacity (DC) and haplotype diversity (HD) of 41 Y-STRs and three Y-InDels were calculated based on the previous report [9]. Likewise, we also calculated the HMP, DC and HD values of different Y-STR sets in the Inner Mongolia Han population. Loci information of different Y-marker sets was given in Supplementary Table S1. Phylogenetic relationship analyses among the studied Han population and other reference populations were dissected *via* multiple methods. Firstly, based on the genetic data of Y-STRs in different Han populations assembled in the Y Chromosome Haplotype Reference Database (YHRD, https://yhrd.org/), *Rst* and *P* values among these Han populations were estimated by the analysis of molecular variance (AMOVA) method [52]. Next, based on pairwise *Rst* values, multidimensional scaling (MDS) analysis and neighbour-joining tree among Han populations in different regions were conducted by SPSS software v18.0 (https://www.ibm.com/products/spss-statistics) and MEGA software v6.0 [53], respectively. Moreover, the same strategies were used to explore genetic differentiations between the Inner Mongolia Han population and other minority groups in China. Genetic data of different minority groups were also obtained from YHRD. Geographical distributions of the studied Han population and other compared populations were plotted by Tableau software (https://www.tableau.com/).

## Results and discussion

### Allele frequencies and GDs of 41 Y-STRs and three Y-InDels

**Figure 1.**
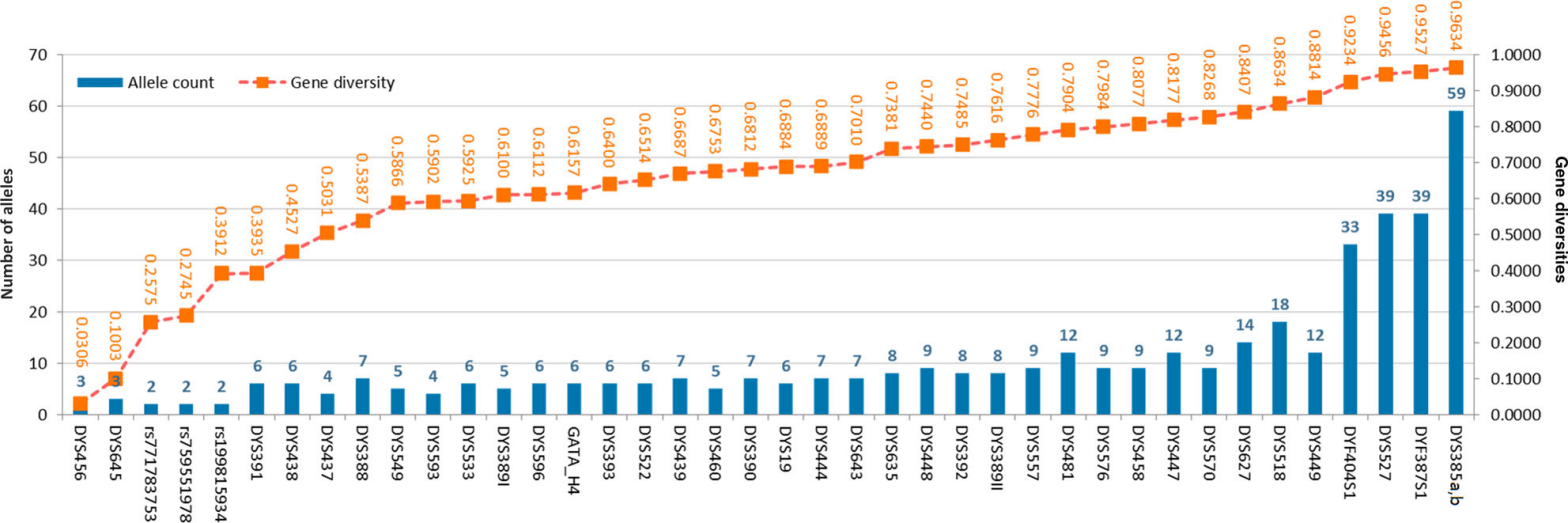
The numbers of alleles and gene diversities of 44 Y-markers in the Inner Mongolia Han population.

Allele distributions and GD values of 41 Y-STRs and three Y-InDels in the Inner Mongolia Han population were shown in Figure 1 and Supplementary Table S2. Totally 2—59 alleles could be observed at these loci, with allelic frequencies distributing from 0.0031 to 0.9846. The least numbers of alleles (two) were seen at three Y-InDel loci; whereas, the maximum was at DYS385a,b loci. For nine rapidly mutating Y-STRs, they showed relatively high numbers of variant alleles (>8). Besides, allele 15 at DYS456 locus displayed extremely high frequency (>0.9800) in the studied Han population in comparison with those in other Han populations [18]. Likewise, we also observed that allele 8 at the DYS645 locus showed high frequency. For the DYS645 locus, similar results could be discerned from previous reports in the Shandong Han population [7] and four minority groups in Hunan Province [34], indicating that allele 8 might have high frequency distributions in Chinese populations. GD values of these 44 Y chromosomal markers distributed from 0.0306 for DYS456 to 0.9634 for DYS385a,b loci. The majority of these loci demonstrated relatively high GD values (>0.5) in the studied Han population; whereas, seven loci had low GD values, especially for DYS456 and DSY645 loci with GD values less than 0.2, implying that these loci possessed relatively low polymorphisms in the studied Han population. Nonetheless, for the 15 new Y-marker sets, 11 novel Y-STRs showed high GD values in the studied Han population, in particular for DYF404S1 and DYS527 loci whose GD values were greater than 0.9, which could enhance the haplotype discrimination power well.

### Variant alleles

Variant alleles commonly include null alleles, intermediate alleles and copy-number variants [8]. In this study, these were eight intermediate alleles in the studied Han population: alleles 18.2, 19.2 and 20.2 at the DYS627 locus, alleles 36.2, 37.2 and 38.2 at the DYS518 locus, allele 12.1 at DYS385a,b loci, and allele 14.2 at DYF404S1 locus. Moreover, we also detected 10 null alleles at three single-copy Y-STRs (DYS389II, DYS447 and DYS448) and three multi-copy Y-STRs (DYF387S1, DYS527 and DYF404S1). Among these Y-STRs with null alleles, the highest numbers of null alleles (5) were observed at the DYS448 locus. A previous study on genetic distributions of 23 Y-STRs in global populations also revealed that the DYS448 locus showed the highest numbers of null alleles, especially in Asian populations (Indian and Pakistan) [8]. Similar phenomena were also observed in Tibetan [54], Shanghai Han [29], and Zhejiang Han populations [55]. Primer binding site mutation or deletion of the targeted region might lead to the occurrence of null alleles. The same results could be observed for the overlapped Y-STRs after these samples were detected again by the Yfiler™ Platinum PCR amplification kit, as shown in Supplementary Figure S1. Therefore, we stated that the deletion of amplification regions might bring about the silence of alleles at these Y-STRs, which needed to be further validated by Sanger sequencing. Furthermore, there was an individual who showed bi-allelic variations at DYS643 and DYS518 loci. Fourteen tri-allelic patterns were observed at DYF387S1, DYS527 and DYF404S1 loci in 11 individuals. Feng et al. [34] assessed the genetic polymorphisms of 50 Y-STRs in Dong, Miao, Tujia and Yao populations, and they also found some diploid variations at single-copy Y-STRs (DYS557, DYS570, DYS439 and DYS576 loci). Additionally, Zhou et al. [29] investigated genetic distributions of 29 Y-STRs in the Shanghai Han population, and they detected tri-allelic patterns at DYF387S1 and DYS385a,b loci, which might be related to the extra copies of these loci in Y chromosome; besides, given that small differences of repeat numbers between alleles at these Y-STRs, they proposed that these extra copies might result from the germline rearrangement of some regions on the Y chromosome. Therefore, we speculated that these additional allele variations might be due to the extra copies of these loci in the Y chromosome, which also needed to be verified *via* Sanger sequencing.

### Haplotype distributions and haplotype resolution comparisons of different Y-marker sets

Haplotype distributions of 44 Y-markers in the Inner Mongolia Han population were presented in Supplementary Table S3. Totally, 319 different haplotypes could be observed in 324 Han individuals with the frequencies ranging from 0.0031 to 0.0062. Among these haplotypes, there were 314 unique haplotypes and five haplotypes observed in two individuals, respectively. The HMP, HD and DC values of 44 Y-markers in the Inner Mongolia Han population were 0.00319, 0.99990, and 0.98457, respectively. The relatively high HD and DC values revealed that these Y-markers could be viewed as a valuable tool for male differentiation in the Inner Mongolia Han population.

**Figure 2.**
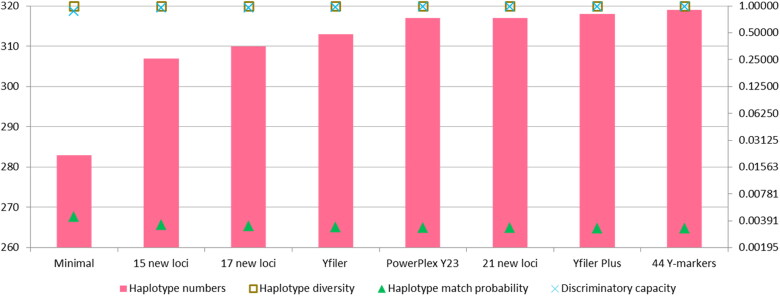
Forensic efficiency comparisons of different Y-STR sets in the Inner Mongolia Han population. Y axis on the left indicates haplotype numbers, and y axis on the right indicates haplotype diversity, haplotype match probability and discriminatory capacity.

Haplotype resolution comparisons of different Y-marker sets in the Inner Mongolia Han population were performed, as shown in Figure 2. Not surprisingly, the least haplotype numbers and lowest HD values were observed at the minimal haplotype; conversely, the most haplotype numbers and highest HD values were at the 44 Y-markers of the SureID PathFinder Plus kit. Similar results could be discerned from the DC distributions among these different Y-marker sets. In comparison with the Yfiler Plus set, there were 310 different haplotypes with HD and DC values of 0.99969 and 0.95679 for 17 new Y-markers. The relatively low HD and DC values of these 17 new Y-markers might result from the limited numbers of Y-markers as compared to Yfiler Plus (27 Y-STRs). Moreover, we found that 21 new Y-markers, not including in the PowerPlex Y23 system, displayed the same haplotype numbers, HD and DC values in comparison with the PowerPlex Y23 system. Since the HD and DC values of the SureID PathFinder Plus kit were higher than those of Yfiler Plus, we thought that the SureID PathFinder Plus kit could provide higher haplotype resolution in the Inner Mongolia Han population than the Yfiler Plus set.

### 3.4. Genetic differentiation analyses of the Inner Mongolia Han population and other reference populations

**Figure 3.**
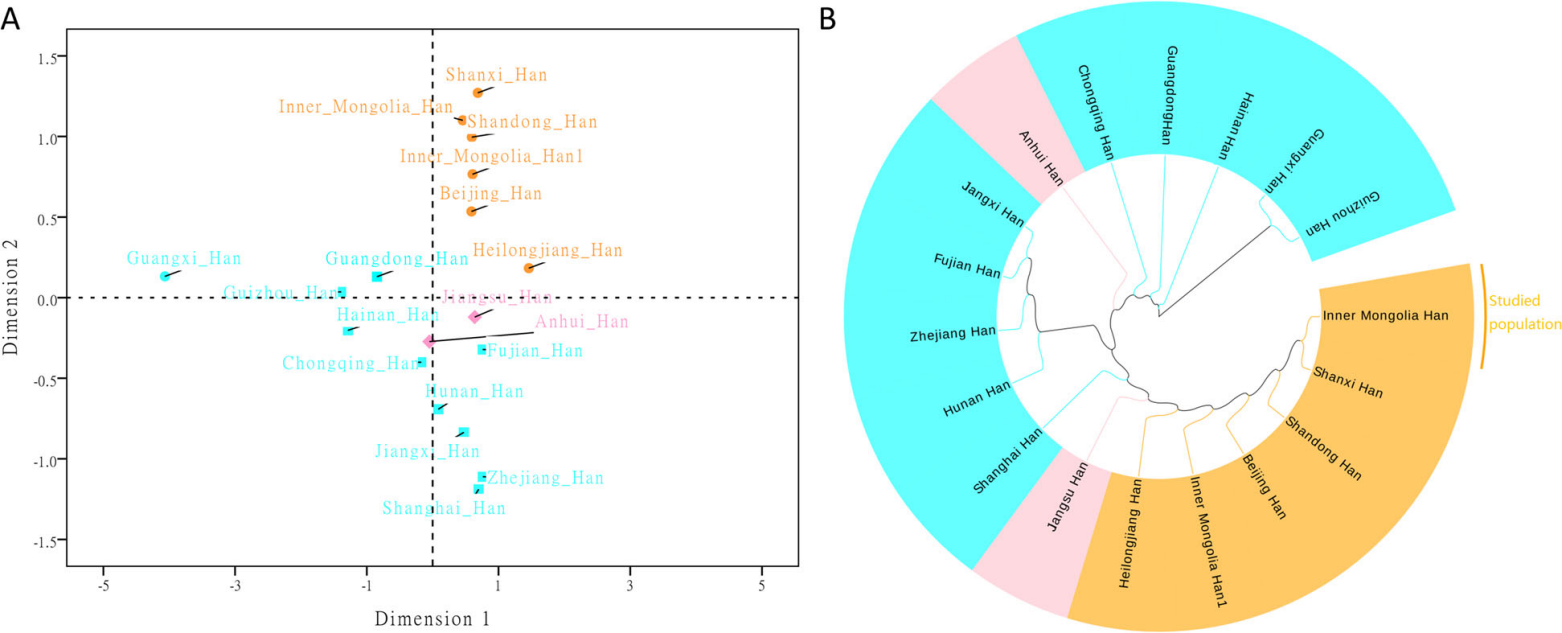
Population genetic analyses of the Inner Mongolia Han population and other Han populations based on 27 Y-STRs of the Yfiler Plus set. (A) Multidimensional scaling (MDS) analysis and (B) Neighbour-joining tree of the Inner Mongolia Han population and other Han populations based on pairwise *Rst* values.

Firstly, we explored genetic relationships among Inner Mongolia Han population and other reported Han populations. The geographical distributions of these Han populations were shown in Supplementary Figure S2. The population information used in this study was presented in Supplementary Table S4. *Rst* distances among these Han populations were provided in Supplementary Table S5. Since low *Rst* values meant low genetic differentiations among populations, some negative *Rst* values were transformed to 0 so as to conduct the following population genetic analyses better. The results revealed that the smallest *Rst* values between the Inner Mongolia Han and Shanxi Han populations were observed, and then Beijing Han, Shandong Han, and Inner Mongolia Han1 populations. Conversely, most of Han populations in other regions including Hainan Han, Guizhou Han, Shanghai Han, Zhejiang Han and Guangxi Han had relatively high *Rst* values with the studied Han population. Besides, *Rst* values between the Inner Mongolia Han and Shanxi Han, Beijing Han and Shandong Han populations were not statistically significant. Next, the MDS plot among these Han populations was conducted based on pairwise *Rst* values, as shown in Figure 3A. The results showed that Northern Han populations, including the studied Han population, located in the first quadrant; most of Southern Han populations positioned in the left and bottom parts. Population distributions in the MDS plot were basically in line with their geographical distributions. Finally, a neighbour-joining tree among these Han populations was constructed, as shown in Figure 3B. The studied Han population firstly clustered with Shanxi Han, Shandong Han, Beijing Han, Inner Mongolia Han1 and Heilongjiang Han populations, and then with other Han populations, showing that the studied Han population had closer genetic affinities with Northern Han populations.

**Figure 4.**
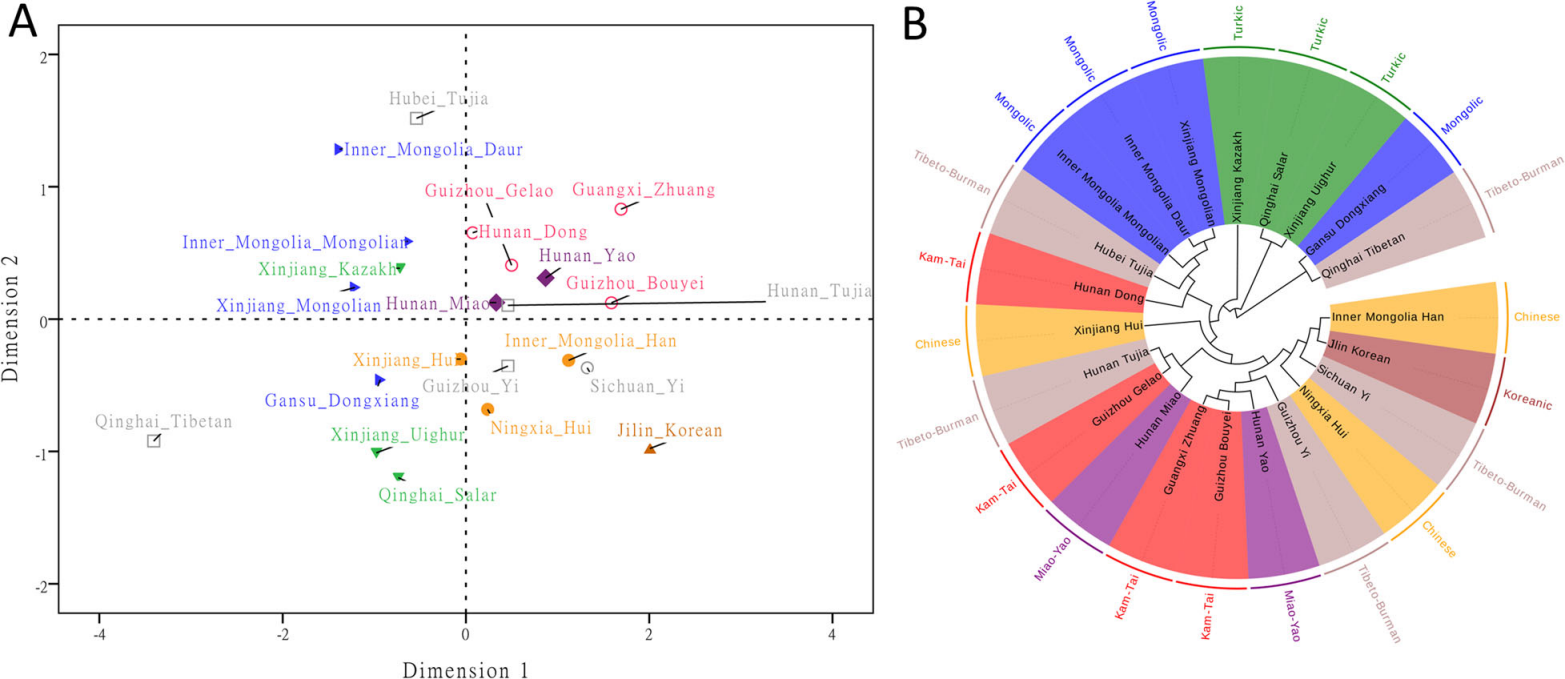
Population genetic analyses of the Inner Mongolia Han population and other minority groups in China based on 27 Y-STRs of the Yfiler Plus set. (A) Multidimensional scaling (MDS) analysis and (B) Neighbour-joining tree of the Inner Mongolia Han population and other minority groups based on pairwise *Rst* values.

Genetic differentiation analyses of the Inner Mongolia Han and other reported minority groups in China were also evaluated, as shown in Figure 4. The geographical locations of these populations were also displayed in Supplementary Figure S2. *Rst* distances between the studied Han population and these minority groups were given in Supplementary Table S6. The results revealed that the studied Han population had relatively small *Rst* values with Ningxia Hui and Sichuan Yi; whereas, it had relatively large *Rst* values with the Inner Mongolia Daur and Qinghai Tibetan groups. Besides, we found that all *Rst* values between Inner Mongolia Han and these minority groups were statistically significant. We also performed the MDS analysis of these populations, as shown in Figure 4A. The distribution patterns of these populations were also in consistent with their geographical distributions: most minority groups in Southern China region situated in the right top corner; most minority groups in Northwest China located in the left part; the studied Han population and some minority groups including Ningxia Hui, Sichuan Yi, Jilin Korean and Guizhou Yi groups located in the right bottom part. Similar population genetic relationships could be discerned from the neighbour-joining tree (Figure 4B). Relatively close genetic relationships among Inner Mongolia Han population and some minority groups might reflect similar Y-haplotype distributions. In previous studies, some researchers also found that Yi and Hui groups showed close relationships with Han populations *via* 30 InDels [56,57]. Therefore, we speculated that Hui and Yi might possess relatively low genetic differentiations with Han populations in comparison with other minority groups, which resulted in close genetic affinities among Hui, Yi and Inner Mongolia Han populations.

There were many population genetic data of different Y-STR sets in Chinese populations [9,19,22,33,40,43], which have been reported till now. However, a few studies on the genetic distributions of Y-STRs in the Inner Mongolia Han population were conducted. In the current study, we firstly provided population data of 44 Y-markers in the Inner Mongolia Han population and evaluated genetic relationships between the studied Han population and other published populations. Population genetic analyses among Han populations in different regions showed that the Inner Mongolia Han population had relatively close genetic relationships with Shanxi Han, Beijing Han and other Northern Han populations. Besides, a north-south gradient among different Han populations could be observed in Figure 3A. Nonetheless, in comparison with different minority groups, low genetic differentiations could be observed among the studied Han population and other Han populations, which might be related to genetic homogenous among different Han populations. In a previous study, Nothnagel et al. [17] comprehensively assessed genetic structure of Chinese male individuals *via* 17 Y-STR loci, and they found that Han populations showed low genetic divergences in comparison with other ethnic groups; furthermore, they also revealed a north-south gradient among Han populations. In a word, the studied Han population had close genetic affinities with Northern Han populations at the point of paternal inheritance. Further research on the genetic distributions of autosomal STRs, mitochondrial genetic markers and ancestry informative markers in the Inner Mongolia Han population and its neighbouring populations should be performed to elucidate genetic relationships among these populations.

## Conclusion

We firstly assessed the genetic distributions of 44 Y-markers in the Inner Mongolia Han population based on the SureID PathFinder Plus kit. Among these loci, most markers showed high genetic polymorphisms in the studied population. The HD and DC values of these 44 Y-markers revealed that the kit could provide the relatively high-resolution haplotype, implying that the kit could be treated as a valuable tool for forensic male differentiation in the studied Han population. Population genetic analyses among the Inner Mongolia Han population and other reported Chinese populations indicated that the studied Han population had closer genetic affinities with Northern Han populations than Southern Han and other ethnic groups.

## Supplementary Material

Supplemental Material

## Data Availability

Genetic data of 44 Y-markers in Inner Mongolia Han population are available from the corresponding author upon request.
