# Supplementary figures and images for: Genetic polymorphisms of 44 Y chromosomal genetic markers in the Inner Mongolia Han population and its genetic relationship analysis with other reference populations
Source: Forensic Sci Res. 2021 Feb 11;7(3):510–7. doi: 10.1080/20961790.2020.1857509 (PMC9639530; doi:10.1080/20961790.2020.1857509)

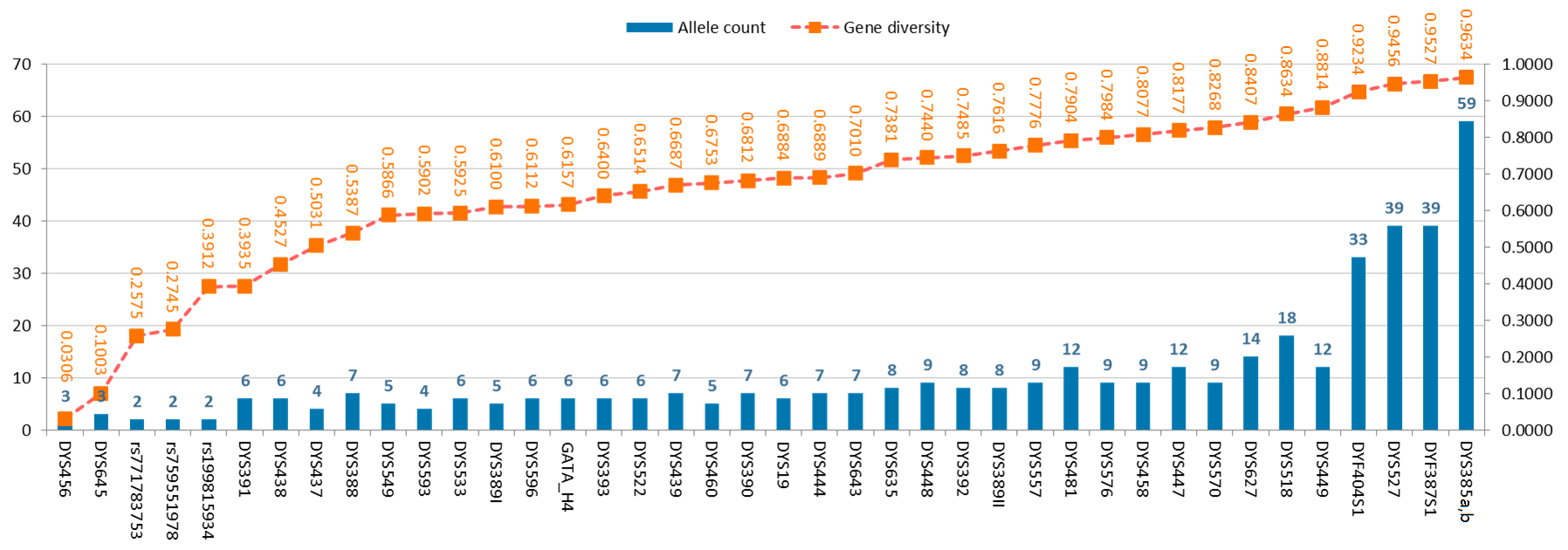

Supplement: Supplemental Material [file TFSR_A_1857509_SM8665.zip › Supplementary Figure 1.tif]

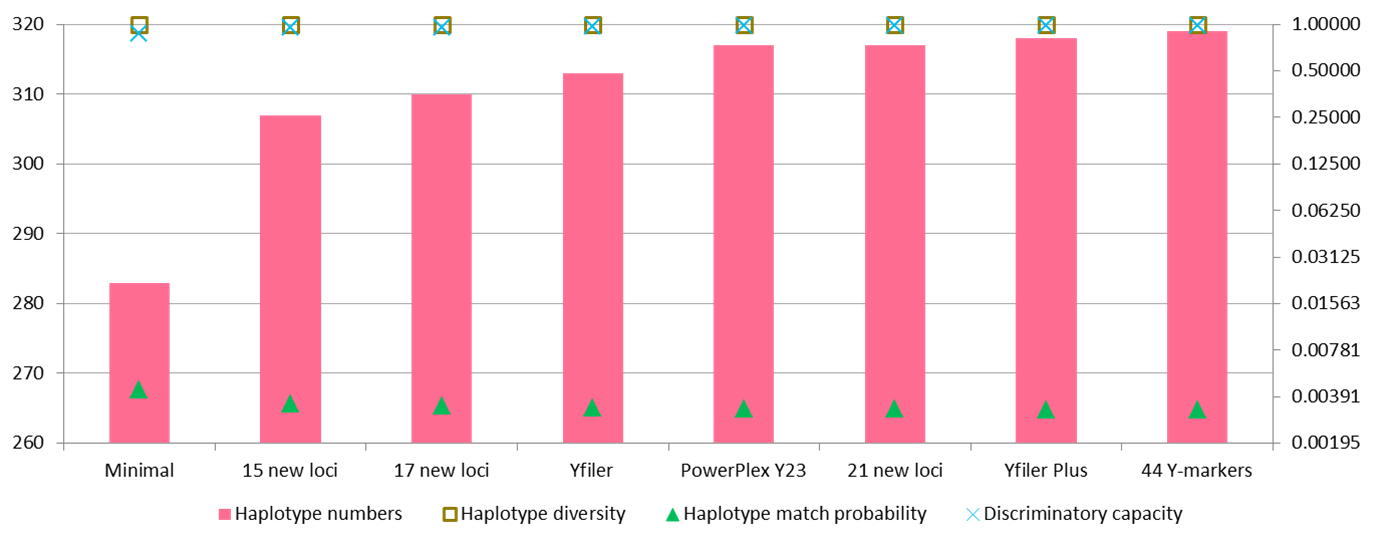

Supplement: Supplemental Material [file TFSR_A_1857509_SM8665.zip › Supplementary Figure 2.tif]
